# Iron Deficiency and Neuroendocrine Regulators of Basal Metabolism, Body Composition and Energy Expenditure in Rats

**DOI:** 10.3390/nu11030631

**Published:** 2019-03-15

**Authors:** Jorge Moreno-Fernandez, Javier Díaz-Castro, María J. M. Alférez, Inmaculada López-Aliaga

**Affiliations:** Department of Physiology (Faculty of Pharmacy, Campus Universitario de Cartuja) and Institute of Nutrition and Food Technology “José Mataix”, University of Granada, E-18071 Granada, Spain; jorgemf@ugr.es (J.M.-F.); malferez@ugr.es (M.J.M.A.); milopez@ugr.es (I.L.-A.)

**Keywords:** ferropenic anaemia, endocrine regulators, body composition, energy expenditure

## Abstract

Although dietary iron is a determinant of iron status in animals, body fat mass has been reported to have an inverse association with iron status in human studies. The goal of this study was to determine the relationship between Fe homeostasis, body composition, energy expenditure and neuroendocrine regulators for severe Fe-deficiency anaemia. Forty male Wistar albino rats recently weaned were divided at random into two groups: the control group was fed the basal diet, AIN-93G diet (normal-Fe) and the anaemic group received a low-Fe diet for 40 days. Neuroendocrine parameters that regulate basal metabolism and appetite (thyroid hormones, ghrelin, glucose-dependent insulinotropic polypeptide (GIP), glucagon, insulin, adrenocorticotropic hormone and corticosterone), body composition, respiratory volumes, energy expenditure, haematological and biochemical were assessed. Total body fat was lower, whereas lean mass, free and total water were higher in the anemic group. O_2_ consumption, CO_2_ production, energy expenditure (EE) and respiratory quotient (RQ) were lower in the Fe-deficient animals. Triiodothyronine and thyroxine hormones decreased, while thyroid-stimulating hormone increased in the anemic group. Circulating levels of ghrelin were lower in the anemic group, while GIP, glucagon, insulin, corticosterone and adrenocorticotropic hormone levels were higher. Fe-deficiency impairs weight gain in the rats, with marked reductions in lean mass and body fat, indicating lower energy stores.

## 1. Introduction

Iron-deficiency Anaemia (IDA) occurs when Fe loss and body’s requirement for iron is not met by dietary sources such that Fe storage of the organism is depleted. This pathological process is characterized by the production of smaller red cells because the concentration of hemoglobin (Hb) is abnormally low [1].

While dietary Fe is a determinant of Fe status, an inverse association between fat mass and Fe status has been previously reported [2]. While studies have shown that increased fat mass might increase the risk of IDA, this is due to the fact that ferritin is an acute-phase protein that is elevated by the low-grade inflammation that occurs when adipose tissues are enlarged [3]. This fact is due that ferritin is an acute-phase protein that may be elevated by the low-grade inflammation that occurs when adipose tissues are enlarged [3]. However, there is scarce information about body composition changes in situation of severe IDA. Given that the burden of chronic diseases is rapidly increasing in developing countries as well as the existence of factors such as deficient diets [4], which influence Fe status and adiposity, it is important to have a good understanding of the relationship between Fe homeostasis and body composition. On the other hand, as the liver is the main Fe storage organ in the organism and it is vital in the regulation of iron homeostasis, the progression of iron depletion during iron deficiency is of great interest.

Metabolic rate is mainly regulated by the central nervous system, which senses energy balance from a wide range of humoral and neural signals, and controls energy intake and basal metabolism [5]. Ghrelin and glucose-dependent insulinotropic polypeptide are two hormones produced mainly by the gut and are secreted from the gastrointestinal tract in response to a meal. Gut hormone concentrations change with weight loss and nutritional deficiencies [6]. Scientific reports have shown that iron deficiency is positively associated with adiposity [7]. In this sense, a decreased concentration of iron, and consecutively haemoglobin, ferritin and decreased level of saturated transferrin, were observed in obese individuals more often than in normal weight subjects [8]. Loss of appetite, retarded growth and development are typical manifestations of the nutritional ferropenic anemia; however, hormonal control for body composition changes during IDA has been not yet fully explained.

Therefore, the current study elucidated the interactions between basal metabolism hormones (ghrelin, GIP, insulin, cortisol, thyroid hormones) haematological status, body composition, and energy expenditure during severe iron deficiency anaemia in young rats.

## 2. Material and Methods

### 2.1. Study Animals

Forty male Wistar albino rats recently weaned at 21 days of life and average weight of 42 g were used. Animals were housing at the Laboratory Animals Unit of the Center of Biomedical Research of the University of Granada, certified as free of pathogens and the animals were housed at conditions of high biological safety, with sanitary and environmental rigorously controlled parameters. The Unit’s housing and handling conditions were approved by the Ethics Committee of the University of Granada (Ref. 11022011) and abide with the Recommendations of the European Community guidelines (Declaration of Helsinki; Directive 2010/63/EU for animal experiments).

### 2.2. Study Design and Diet

After weaning, rats were placed on an experimental period (EP) of 40 days and randomly divided into two groups, a control group fed AIN-93G diet [9] and an anaemic group, receiving the same diet, but with a low-Fe content (5 mg Fe/kg diet) [10]. Nutrient composition of the experimental diet is shown in Table 1. The analysis of content of Fe in the diet (mg/kg) was as follows: 44.82 for normal-Fe diet and 6.21 for low-Fe diet. Diet intake was controlled, pair feeding all the animals (80% of the average intake) and deionized water was available ad libitum. Animals were housed in individual metabolic cages in an environmentally controlled room on a 12 light/12 dark cycle (9:00 a.m.–9:00 p.m.), at 22 ± 1 °C and humidity of 55–65%. Feed and mineral-free water were provided ad libitum. At the end of EP, whole body composition and respiratory volumes and flows and energy expenditure were assessed. At the end of the EP, whole body composition and respiratory volumes and flows and energy expenditure were assessed as described in 2.10–2.12. After fasting overnight, animals were weighed and anesthetized by intraperitoneal injection of sodium pentobarbital (5 mg/100 g body weight) (Sigma, St Louis, MO, USA). After midline laparotomy, the rats were bled from the abdominal aorta, one aliquot of blood was collected in tubes with EDTA anticoagulant for haematological analysis. Serum was separated from the EDTA-free aliquot by centrifugation at 1500× *g* for 10 min at a 4 °C and stored at −80 °C to measure ferritin, transferrin saturation, total iron binding capacity (TIBC) and biochemical parameters. Liver was removed, rinsed in ice-cold normal saline solution (0.9%, *w*/*v*, NaCl), immediately weighed and stored at −20 °C for Fe analysis.

### 2.3. Dry Matter

Determination of water content (in diet and liver) was carried out by drying the material to a constant weight at 105 + 2 °C in an oven (~48 h).

### 2.4. Iron Determination

The diet and liver samples were previously mineralized by wet method in a sand bath (J.R. Selecta, Barcelona, Spain); the samples were placed in a resistant flask and dissolved using nitric acid followed by a mixture of HNO_3_:HClO_4_ (69%:70%, *v*/*v*; Merck KGaA, Darmstadt, Germany; ratio 1:4, *v*/*v*) until the total elimination of organic matter. Finally, the samples were diluted with bidistilled ultrapure Milli-Q water. Fe analysis was undertaken using an Optima 8300 (PerkinElmer Inc. Waltham, MA, USA) inductively coupled plasma-optical emission spectrometer (ICP-OES) with a segmented-array charge-coupled Device (SCD) high-performance detector. Fe was analysed in liver and diets according to compatibility under optimised set of conditions. For the calibration of the apparatus, multi elemental Astasol calibration solutions (Analytika, Khodlova, Prague) were used. For the calibration curve, the following working dilutions of the analytical standard were prepared: 0.1, 0.5, 1.0, 10, 50 mg/L. An internal standard solution of 10 mg L^−1^ was used after each series of five samples. The acceptable result was assessed as 10%. The samples were measured in three replicates.

### 2.5. Haematological Test

Haemoglobin (Hb) concentration, red blood cells (RBCs), haematocrit, mean corpuscular volume (MCV), mean corpuscular Hb (MCH), mean corpuscular Hb concentration (MCHC), red cell distribution width (RDW), platelets, white blood cells (WBCs) and lymphocytes, of fresh blood samples were measured using an automated hematology analyzer Mythic 22CT (C2 Diagnostics, Grabels, France).

### 2.6. Serum Iron, Total Iron Binding Capacity and Transferrin Saturation

Serum iron and total iron binding capacity (TIBC) were determined using a quantitative colorimetric enzymatic assay (Sigma Diagnostics Iron and Total Iron- Binding Capacity reagents, Sigma Diagnostics, St Louis, MO, USA). The absorbance was read at 550 nm on a microplate reader (Bio-Rad Laboratories Inc., CA, USA). Transferrin saturation percentage was calculated by dividing serum iron by TIBC, and then multiplying by 100.

### 2.7. Serum Ferritin

Serum ferritin concentration was determined using the Rat Ferritin ELISA Kit (Biovendor Gmbh, Heidelberg, Germany). The absorbance of the reaction mixtures was read in a microplate plate reader at 450 nm (reference 650 nm) using a Bio-Rad microplate reader (Bio-Rad Laboratories Inc., CA, USA). The intensity of the color was inversely proportional to the serum ferritin concentration.

### 2.8. Serum Hepcidin

Hepcidin-25 concentration was determined using a DRG ELISA Kit (DRG Instruments GmbH, Marburg, Germany). The microtiter wells were coated with a monoclonal (mouse) antibody directed toward an antigenic site of the hepcidin-25 molecule. Endogenous hepcidin-25 of a sample competed with a hepcidin-25-biotin conjugate for binding to the coated antibody. After incubation, the unbound conjugate was washed off and a streptavidin-peroxidase enzyme complex was added to each well. Substrate was added to detect antigen–antibody–enzyme complex and development of blue color. The microplate was read at 450 nm on a microplate reader (Bio-Rad Laboratories Inc.) and the intensity of color developed was reverse proportional to the concentration of hepcidin in the sample. Results were expressed in nanograms per milliliter of serum.

### 2.9. Biochemical Parameters

Serum total protein, albumin, total cholesterol, LDL-cholesterol, triglycerides, glucose, transaminases (AST and ALT), bilirubin, urea, creatinine, amylase, cortisol and creatine kinase-MB were measured by standard colorimetric and enzymatic methods, using a BS-200 Chemistry Analyzer (Shenzhen Mindray Bio-Medical Electronics Co. Ltd., Shenzhen, China). Two replicate samples were analyzed for measure each parameter, and the averages in each paired data were determined.

### 2.10. Assessment of Body Composition

Whole body composition (fat and lean tissues) was determined using quantitative magnetic resonance (QMR) with an Echo MRI Analyzer system by Echo Medical Systems (Houston, TX, USA) [11]. All QMR measurements were made during the light phase (09:00 A.M.–6:00 P.M.). Scans were performed by placing animals into a thin-walled plastic cylinder (3 mm thick, 6.5 cm inner diameter), with a cylindrical plastic insert added to limit movement. While in the tube, animals were briefly subjected to a low-intensity (0.05 Tesla) electromagnetic field to measure fat, lean mass, free water, and total body water. Briefly, this system generates a signal that modifies the spin patterns of hydrogen atoms within the subject, and uses an algorithm to evaluate the four components measured—fat mass, lean muscle mass equivalent, total body water, and free water. QMR scans were performed with accumulation times of 2 min.

### 2.11. Respiratory Gas Collection, Energy Expenditure Calculation and Analysis

The instruments used for measurement of the respiratory quotient in the rats consisted of acrylic metabolic chambers, gas analyzers (model LE 405 Gas Analyzer Panlab Technology for Bioresearch, Madrid, Spain), and a switching system (model L 400 Air Supply and Switching, Panlab Technology for Bioresearch). Prior to data collection, the system was calibrated using certified gas cylinders (Gilmore Liquid Air Co., South El Monte, CA, USA) containing 50% O_2_/5% CO_2_/45% N_2_ (high point calibration) and 20% O_2_/0% CO_2_/80% N_2_ (low point calibration) per manufacturer’s instructions. A clear, metabolic chamber, containing a small amount of bedding material, was placed on a over the Pyrex pan. The ambient chamber temperature was maintained at 22 ± 2 °C. All the weights were entered into the derived inlet flow rate equation to estimate the appropriate value. Individual rats were placed in the metabolic chamber and allowed to acclimate for 15 min prior to data collection. During this period, room air was allowed to flow through the chamber. After acclimation, respiratory gas analysis was carried out utilizing Metabolism H software (Panlab, S.L., Barcelona, Spain) running on a HP Compaq Pentium 4 computer (Hewlett-Packard Company, Palo Alto, CA, USA). The data collection paradigm consisted of (1) a 15-min acclimation period during which time room air was flowing through the metabolic chamber, (2) a 1-min room air reference sampling period, and (3) a 10-min chamber air sampling period during 24 h. This paradigm yielded enough time for the small volumes of gases exchanged by the rat to reach equilibrium within the chamber, accommodating the inherently slow sampling process associated with low inlet and sampling flow rates. The mean values of VO_2_, VCO_2_, respiratory quotient (RQ) and daily energy expenditure (EE) over the 10-min chamber sampling period were then calculated by the Metabolism H software (Harvard Apparatus, Massachusetts, MO, USA). The total time the rats spent on the system was 24 h each. Metabolic data were collected from individual rats of each experimental group and were analyzed during the day (tested between 9:00 a.m. and 9:00 p.m., and night (tested between 9:00 p.m. and 9:00 a.m.) to explore the possible influence of time (during the light phase) on the studied metabolic parameters. By utilizing an open-circuit system that pulled air into the chamber under slightly negative pressure to maintain a constant flow rate, the influence of the rat’s physical activity on internal chamber pressure (and thus on the ability to measure true VO_2_) was avoided.

### 2.12. Thyroid Hormones, Ghrelin, Glucose-Dependent Insulinotropic Polypeptide, Glucagon, Insulin, Adrenocorticotropic Hormone and Corticosterone Measurement

Triiodothyronine (T_3_), thyroxine (T_4_) and thyroid-stimulating hormone (TSH), were determined using the RTHYMAG-30K Milliplex MAP Rat Thyroid Magnetic Bead Panel; ghrelin (active), GIP (total), glucagon and insulin, were determined using the RMHMAG-84K Milliplex MAP Rat Metabolic Hormone Magnetic Bead Panel; adrenocorticotropic hormone (ACTH) and corticosterone plasma levels were determined using the RSHMAG-69K Milliplex MAP Rat Stress Hormone Magnetic Bead Panel (Millipore Corporation, MO, Massachusetts, USA), based on immunoassays on the surface of fluorescent-coded beads (microspheres), following the specifications of the manufacturer (50 events per bead, 50 µL sample, gate settings: 8000–15,000, time out 60 s, bead set: 34). Plate was read on LABScan 100 analyzer (Luminex Corporation, Austin, TX, USA) with xPONENT software (MO, Massachusetts, USA) for data acquisition. Average values for each set of duplicate samples or standards were within 15% of the mean. Thyroid hormones, ghrelin, GIP, glucagon, insulin ACTH and corticosterone concentrations in plasma samples were determined by comparing the mean of duplicate samples with the standard curve for each assay.

### 2.13. Statistical Analysis

Statistical analysis was performed with SPSS version 20.0 (SPSS Inc., Chicago, IL, USA) software package. Data were expressed as means ± standard error of the mean (SEM). Prior to perform any statistical analysis, all variables were checked to assess the equality of variances (homogeneity of variance) using the Levene’s test. Student’s *t*-test for independent samples was used to check the difference in mean between control vs. anaemic groups. A value of *p* < 0.05 was considered statistically significant.

## 3. Results

### 3.1. Haematological and Bichemical Parameters

After Fe deprivation, differences in were found in all the haematological parameters analysed on both experimental groups. Most of the haematological parameters were drastically low (*p* < 0.001): Hb concentration, RBCs, haematocrit, MCV, MCH, MCHC serum Fe, transferrin saturation, serum ferritin, and serum hepcidin. On the other hand, platelets, RDW and TIBC increased markedly (*p* < 0.001), while no changes were recorded in WBCs (Table 2). All of these results indicate that the Fe-deficiency anaemia was experimentally induced in the rats. With regard to the biochemical parameters, albumin, total cholesterol, triglycerides, glucose, AST, ALT, bilirubin, urea, amylase and cortisol increased markedly in the Fe-deficient group in comparison with the control group (*p* < 0.001) (Table 2).

### 3.2. Hepatosomatic Index

Body weight was lower in anaemic animals as compared to the control animals (*p* < 0.001), and liver weight did not change significantly. As a consequence, the hepatosomatic index (ratio of liver weight to body weight) was higher in the Fe-deficient animals (*p* < 0.05). The differences in the hepatic Fe content on day 40 of the study were markedly pronounced (*p* < 0.001) and directly correlated with the Fe-restriction in the diet (Table 3).

### 3.3. Body Composition

Marked differences were found in body composition parameters between both experimental groups. Body fat was lower in the anaemic group compared with the control group (*p* < 0.001). In contrast, lean mass, free water and total water were higher in the Fe-deficient group (*p* < 0.01 for lean mass and *p* < 0.001 for free and total water) (Table 4).

### 3.4. Respiratory Volumes and Flows, Energy Expenditure, O_2_ Consumption

**Figure 1:**
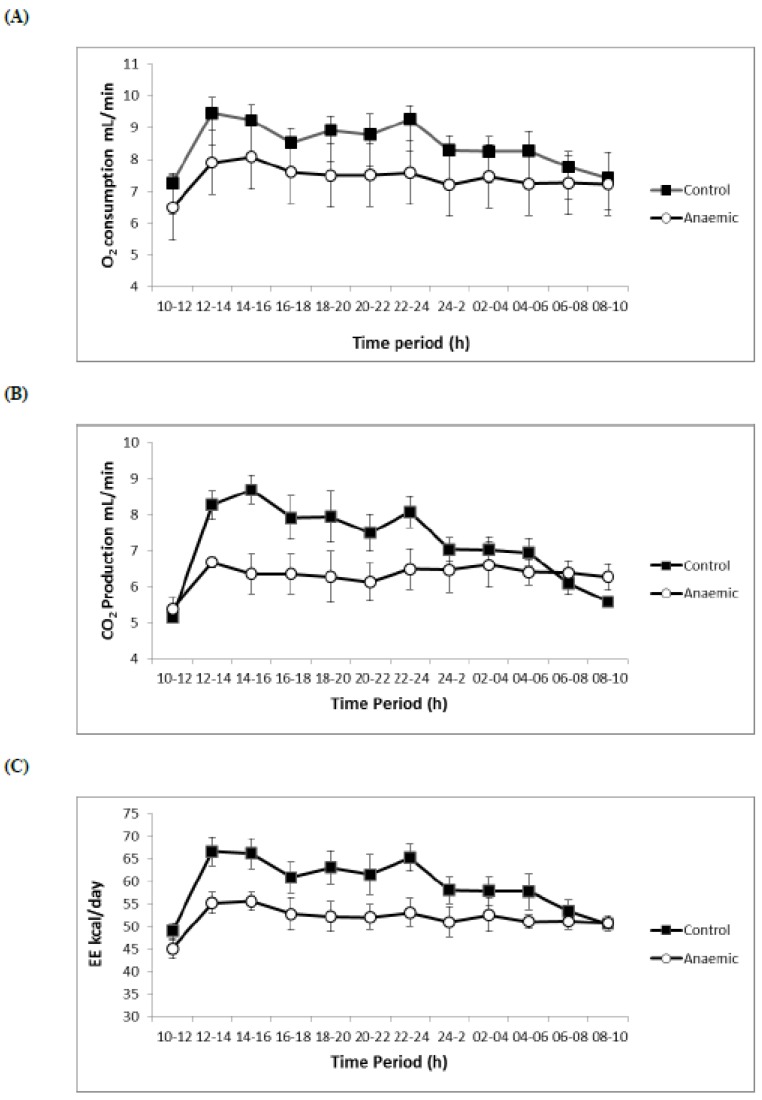
Effect of time of day and anaemia on metabolic parameters. Rats were surveyed every 10 min at an ambient temperature of 22 ± 2 °C. All data are expressed as means ± SEM. (**A**) oxygen consumption (VO_2_), (**B**) carbon dioxide production (VCO_2_), (**C**) Energy expenditure (EE).

With regard to respiratory volumes and flows, EE, O_2_ consumption was lower in the Fe-deficient rats in both periods (day and night) (*p* < 0.001), CO_2_ production was also lower in the anaemic group (*p* < 0.001 during the day and *p* < 0.01 during the night), EE decreased in the Fe-deficient group (*p* < 0.001 in both periods) and RQ also decreased (*p* < 0.01 in both periods) (Table 5, Figure 1).

### 3.5. Neuroendocrine Regulators of Basal Metabolism

Endocrine regulators of basal metabolism also showed differences between both experimental groups (Table 6). Triiodothyronine (T_3_) and thyroxine (T_4_) decreased (*p* < 0.001) while thyroid-stimulating hormone (TSH) increased (*p* < 0.001) in the anemic group (Table 6). Ghrelin decreased (*p* < 0.001) in the anemic group, while GIP, glucagon, insulin, corticosterone and adrenocorticotropic hormone (ACTH) increased in the Fe-deficient group (*p* < 0.01 for GIP, glucagon and insulin; *p* < 0.001 for corticosterone and ACTH.

## 4. Discussion

The study investigated changes in basal metabolism hormones, body composition, and energy expenditure during severe iron deficiency anaemia in young rats. Transferrin saturation, the most widely used screening measurement in Fe-deficiency anaemia, is low in this pathology [12,13], resulting in agreement with those obtained in the current study. MCV is a key red-cell marker for detecting Fe-deficiency anaemia in erythrocytes [14]. Serum ferritin has been a routine laboratory measure of Fe status because it is a well-standardized measurement for identifying Fe-deficiency because its concentration is directly proportional to body Fe stores [15]. On the other hand, RDW increased significantly in IDA because circulating Fe did not reach the bone marrow and red cells were deprived from Fe until this size was elevated. In IDA, Fe is not present in the enterocytes and macrophages because it is pumped from enterocytes across ferroportin 1 (FPN1) channel because hepcidin is low and therefore unable to prevent Fe releasing into the plasma [16]. Therefore, in light of all that is mentioned above, we can report that the body stores were significantly depleted after dietary Fe restriction during 40 days.

Bilirubin is the terminal product of heme metabolism, and total serum bilirubin levels are increased in hepatocellular injury, intra and extra hepatic biliary duct obstructions, intravascular and extravascular hemolysis. In the current study, bilirubin was increased and AST and ALT activities were also found elevated in IDA, which suggest necrosis liver function stress and impairment [17]. In our study, Fe-deficiency increased cortisol secretion, a fact that would increase energy expenditure and suppress body fat accumulation [12]. This increase in cortisol secretion is also associated with enhanced lipolysis, plasma triglyceride and cholesterol [18], supporting the changes in body composition of the anaemic group. With regard to glucose, increase in the Fe-deficient group, this can be attributed to the hyperglycemic effect of cortisol.

The differences in the on anaemic group reveals that hepatic Fe content is depleted and a severe degree of Fe-deficiency has been reached, induced by consuming a low-Fe diet for 40 days. The body weight is lower in anaemic animals, and hepatic weight also descended slightly. As a consequence, the hepatosomatic index (ratio liver weight/ body weight) is higher in the Fe-deficient animals. The lower Fe content recorded in liver in situation of IDA can be due to the decrease of the hepcidin recorded in the current study, a fact that is in agreement with the results reported previously by [19]. Hepcidin is a hepatic peptide, which has been identified as the master regulator of Fe metabolism [20]. Studies of Fe deficiency in hepcidin KO mice suggest that this peptide intervenes in regulating the storage of the mineral (attenuating both the intestinal Fe absorption and the liberation of the Fe of the macrophages) [21].

On the other hand, a low expression of hepcidin during Fe overload suggests that this protein is also a key factor in the erythroid regulation [22]. The diminished expression of hepcidin in response to the hypoxia induced by the Fe-deficiency, and its increased expression in mice and humans suffering with inflammation processes suggests that this hormone also takes part in the hypoxia homeostatic response and in the pathways of inflammatory regulators [23]. Hepcidin also controls the Fe levels directly acting reciprocally with the FPN1, driving to the internalization and degradation of FPN1 when levels of Fe are high, consequently blocking the liberation of the Fe in the sites of storage: hepatocytes, enterocytes and macrophages [24].

During Fe-deficiency, several regulatory factors of the hepcidin are impaired (the erythropoietic demand increases due to the decrease of the hematologic parameters, there is a minor supply of oxygen to the tissues and the body stores are depleted, being the Fe metabolism altered), facts that lead to a decrease of the above mentioned hormone, which will be translated in a minor interaction with the FPN1 [25], avoiding its internalization and degradation, therefore increasing the outflow of Fe^2+^ from the hepatocytes and its storage diminishes consistently in the above mentioned organ.

On the other hand, an Fe-restrictive diet causes a severe iron deficient anaemia enough to impair the body weight, which results in agreement with those reported by other authors [26], and this can be attributed to the lower levels of thyroid hormones found in this pathology in the current study and in previous reports [27]. Fe deficiency–induced alterations in the central nervous system impair thermoregulatory responses that induce the lower thyroid hormone response and the overall failure to thermoregulate that characterize Fe-deficient rats, diminishing basal metabolic rate and weight gain. TSH increased as a compensatory mechanism to enhance T_3_ and T_4_ production, diminished by the Fe-deficiency. In addition, our group reported that severe IDA has a significant impact upon bone turnover and mass, decreasing bone formation and increasing its resorption [28], thus implying delayed skeletal development. This can also explain reduced weight. Additionally, impairment of metabolic energy output, and cellular respiration of rats caused by Fe deficiency could be also responsible for the reduced body weight.

After Fe deprivation in the anemic group, cortisol levels increased significantly (Table 2). This can possibly be explained by the lower activity of monoaminooxidase in IDA, and the low level of aldehyde oxidase activity. These enzymatic changes induce an increase in the endogenous levels of circulating catecholamines, which enhance the release rate of adrenocorticotrophic hormone, producing in turn an increase in glucocorticoids output [12]. In addition, anemia can be considered a pro-inflammatory state, arising in part from a defect in the normal compensatory production of erythropoietin in response to a declining hemoglobin concentration [29]. In addition, inflammatory cytokines may negatively influence Fe absorption and recycling, thereby interfering with hemoglobin synthesis [30]. Therefore, the increase in corticosterone could be a compensatory mechanism to alleviate, at least partly, the anemia-induced inflammatory state because Glucocorticoids protect endothelial and epithelial cells from stress-induced apoptosis, exhibiting potent anti-inflammatory effects [31].

Ghrelin levels were decreased in anemia situation. In the presence of catabolism, plasma total ghrelin levels are increased, suggesting that, in that case, ghrelin does not increase food intake and/or anabolism under these circumstances. In addition, it is currently unknown whether administration of additional ghrelin in these conditions may reduce the development of cachexia [32]. Ghrelin levels detected in the current study decreased in parallel to Fe stores. Therefore, there is a decrease in appetite during IDA, leading to a reduction in body weight of the rats. The reduced ghrelin secretion could be a compensatory mechanism in reaction to the metabolic consequences of increased corticosterone secretion (Table 2) and induced catabolism [33].

Insulin was higher in the IDA group, together with an increase in glucagon, which could be due to the increased corticosterone secretion. The increase in insulin production has been previously reported in IDA [34]. These authors reported that, although basal glucose was significantly elevated in rats with IDA, there was also an increase in peripheral insulin responsiveness, a fact that could be beneficial in keeping glucose levels within a physiological range, in spite of the high corticosterone secretion. However, in spite the increased insulin responsiveness which could be beneficial to the higher insulin and high glucagon output, an increase in basal glucose was observed (Table 2).

Upon dietary ingestion, glucose-dependent insulinotropic polypeptide (GIP) hormones are secreted by K cells from the upper small intestine and they induce pancreatic cells to release insulin [35]. Consequently, the higher insulin rate secretion found in the current study can be explained by an increase in GIP secretion. In addition, studies in rodent models indicate that regulation of fat metabolism is an important physiological function of GIP that induces glucose uptake, activity of lipoprotein lipase, and accumulation of triglycerides by adipocytes [36], explaining the increase in total cholesterol and triglycerides found in the anemic rats (Table 2).

There are controversies in several studies, which reported associations between fat mass and various markers of Fe status [2,6,7,8,37,38]. In these studies, the direction of the association depends on the Fe biomarker (inverse for serum Fe, positive for serum ferritin) [2,37,38]. The report of Yanoff [38], indicating an association between obesity and serum ferritin concentration, suggests that the degree of fat mass might play a major role in predicting body Fe status. In other words, an association might be observed in subjects with a higher percent body fat but not in those in the normal range of adiposity, as occurs in the current study in which the animals are non-obese. Based on the study by Yanoff et al. [38], the hypoferremia observed during obesity appears to be explained both by true Fe deficiency and by inflammatory-mediated functional Fe deficiency.

In the present study, decreases in the body weight and adipose tissue mass were recorded in Fe-deficient rats, without changing the energy intake. In terms of energy intake, cortisol secreted during sympathetic nervous system action plays a key role. It is well known that normal digestion and absorption of dietary fat requires the action of pancreatic lipase. Because the sympathetic nervous system regulates, to a large extent, thermogenesis and fat oxidation, and cortisol release provides an explanation to a fat mass management in the situation of anemia [39,40].

Another important finding in this study was that body composition analysis showed increased free water in Fe-deficient animals, indicating edema and meaning a severe hemodynamic alteration together with overload of rennin–angiotensin–aldosterone system, which is unable to maintain the distribution of water between the intra and extracellular spaces [41].

With regard to the low O_2_ consumption, CO_2_ production and EE in the anaemic group, since in an Fe-deficiency situation, the Hb and RBC count drastically diminishes the supply of O_2_ to the cells, which limits itself considerably. The lower O_2_ consumption recorded in the current study affects in a negative way to the ATP synthesis, limiting and reducing the EE and impairing weight gain. These results agree with the findings of Schneider et al. [42], who reported that low levels of Hb significantly harm the weight gain, explaining therefore the association found in the present study between the minor weight and the Fe-deficiency. A limitation of the study is that statistical power could be impaired by fewer numbers (20 for each group), although, in many similar studies, the numbers were even lower (10 for each group), and numbers could be increased in future studies. Another limitation of the study is that, although animals are supplied healthy and pathongens-free, markers for infection and inflammation were not determined in serum and tissue samples of the mice used in the study.

## 5. Conclusions

In conclusion, the severe induced Fe-deficiency provokes an impairment of haematological and biochemical parameters and a depletion of the hepatic Fe levels together with a slight decrease of the liver size that negatively impacts the weight gain of animals in growth, since the hypoxia induced by the lack of Fe limits ATP production. Fe-deficiency impairs body weight and it is possibly due to the lower levels of thyroid hormones also observed in the current study. The increase in TSH recorded would be a compensatory mechanism to induce T_3_ and T_4_ production, diminished by the Fe-deficiency. After Fe deprivation in the anemic group, an increase in the cortisol levels was also recorded. Ghrelin levels decreased in an anemic situation, explaining the cachexia induced by Fe-deficiency and leading to reductions of lean mass. GIP increased during Fe deficiency leading to a higher insulin rate secretion. In addition, IDA showed marked reductions lean mass and reduced body fat, indicating reduced energy stores. Body weight is a composite index of both the lean and fat mass, and it cannot totally account for the growth deficit in rats suffering from Fe-deficiency because of the difference in relative proportions of lean and fat mass in the rat.

## Figures and Tables

**Table 1:** Composition of the experimental diets.

Component	g/Kg Diet (Dry Weight)
Protein (casein)	210
Fat (olive oil)	100
Fibre (micronized cellulose)	50
Mineral supplement ^a^	35
Vitamin supplement ^b^	10
Choline chloride	2
Wheat starch	491
Sucrose	100
L-cystine	2
Energy (kJ/kg)	17940

^a^ During the experimental period (EP), the mineral premix was prepared according to the recommendations of the AIN-93 G Diet [9] for control rats (normal-Fe: 45 mg/kg diet) and for anaemic rats (low-Fe: 5 mg/kg diet) [10]. ^b^ The vitamin premix were prepared according to the recommendations of the AIN-93 G Diet [9] for growing rats.

**Table 2:** Haematological and biochemical parameters of control and anaemic rats.

*Haematological Parameters*	Normal-Fe Control Group (*n* = 20)	Low-Fe Anaemic Group (*n* = 20)
Hb concentration (g/L)	138.83 ± 3.19	60.36 ± 3.22 *
RBCs (10^12^/L)	7.05 ± 0.19	3.02 ± 0.24 *
Haematocrit (%)	39.87 ± 1.14	11.61 ± 1.32 *
MCV (fL)	56.40 ± 0.55	38.26 ± 0.37 *
MCH (pg)	19.91 ± 0.14	14.32 ± 0.68 *
MCHC (g/dL)	35.24 ± 0.36	30.88 ± 0.87 *
RDW (%)	16.67 ± 0.34	19.31 ± 0.42 *
Platelets (10^9^/L)	745 ± 73.19	2251 ± 118 *
WBCs (10^9^/L)	8.88 ± 0.38	8.26 ± 0.97
Lymphocytes (10^6^/mL)	8.01 ± 0.61	5.88 ± 0.85 *
Serum Fe (µg/L)	1350 ± 106	598 ± 57.01 *
TIBC (µg/L)	2756 ± 197	18235± 676 *
Transferrin saturation (%)	48.55 ± 6.49	3.75 ± 0.41 *
Serum ferritin (µg/L)	81.45 ± 2.37	49.12 ± 1.48 *
Serum hepcidin (ng/mL)	16.98 ± 0.45	12.51 ± 0.62 *
*Biochemical parameters*		
Total protein (g/dL)	4.97 ± 0.15	5.27 ± 0.13
Albumin (g/dL)	2.84 ± 0.04	3.34 ± 0.12 *
Total cholesterol (mg/dL)	88.92 ± 7.45	109.27 ± 9.69 *
LDL-cholesterol (U/L)	2815 ± 444	2829 ± 446
Triglycerides (mg/dL)	73.92 ± 3.37	217.52 ± 28.46 *
Glucose (mg/dL)	69.61 ± 3.97	86.13 ± 3.68 *
AST (IU/L)	103.58 ± 8.93	228.04 ± 18.45 *
ALT (IU/L)	24.57 ± 1.16	52.28 ± 2.73 *
Bilirubin (mg/dL)	0.81 ± 0.09	1.25 ± 0.13 *
Urea (mg/dL)	33.32 ± 1.77	42.47 ±2.01 *
Creatinine (mg/dL)	0.048 ± 0.017	0.043 ± 0.015
Amilase (U/L)	76.40 ± 5.16	379.35 ± 16.81 *
Cortisol (µg/L)	21.51± 1.07	42.21± 1.88 *
Creatine kinase-MB (U/L)	1531 ± 112	1391 ± 112

Mean values ± SEM. Hb haemoglobin, RBCs red blood cells, MCV mean corpuscular volume, MCH mean corpuscular Hb, MCHC mean corpuscular Hb concentration, RDW red cell distribution width, WBCs white blood cells, TIBC total Fe-binding capacity, LDL-cholesterol low-density lipoprotein cholesterol, AST aspartate aminotransferase, ALT alanine aminotransferase. * Significantly different (*p* < 0.001) from the control group by Student’s *t*-test.

**Table 3:** Hepatosomatic index and liver Fe content in control and anemic rats.

	Normal-Fe Control Group (*n* = 20)	Low-Fe Anaemic Group (*n* = 20)
Body weight (g)	242.7 ± 4.6	200.8 ± 2.6 **
Liver weight (g)	6.415 ± 0.26	6.032 ± 0.34
Liver weight/body weight (%)	2.59 ± 0.06	2.83 ± 0.09 *
Liver Fe content (µg/g dry weight)	609.26 ± 34.12	424.12 ± 23.10 **

Mean values ± SEM. * Significantly different (*p* < 0.05) from the control group by Student’s *t*-test. ** Significantly different (*p* < 0.001) from the control group by Student’s *t*-test.

**Table 4:** Body composition in control and anemic rats.

	Normal-Fe Control Group (*n* = 20)	Low-Fe Anaemic Group (*n* = 20)
Fat (%)	7.29 ± 0.36	6.38 ± 0.41 **
Fat (g) ^1^	22.20 ± 2.35	15.62 ±1.57 **
Lean mass (%)	90.63 ± 0.47	92.51 ± 0.63 *
Lean mass (g) ^1^	263.52 ± 4.80	242.17 ± 3.69 *
Free water (%)	0.42 ± 0.04	0.76 ± 0.09 *
Free water (g) ^1^	1.24 ± 0.21	1.12 ± 0.21 *
Total water (%)	76.79 ± 0.57	79.89 ± 0.45 **
Total water (g) ^1^	223.31 ± 4.09	206.47 ± 3.09 **

Mean values ± SEM. * Significantly different (*p* < 0.01) from the control group by Student’s *t*-test. ** Significantly different (*p* < 0.001) from the control group by Student’s *t*-test. ^1^ Includes water contained in the tissue.

**Table 5:** O_2_ consumption, CO_2_ production, energy expenditure and respiratory quotient in control and anaemic rats.

	Normal-Fe Control Group (*n* = 20)	Low-Fe Anaemic Group (*n* = 20)
*Day period*		
VO_2_ (mL/min)	8.86 ± 0.15	7.43 ± 0.09 **
VCO_2_ (mL/min)	7.91 ± 0.20	6.07± 0.32 **
EE (kcal/day)	62.74 ± 0.95	51.59 ± 0.97 **
RQ	0.89 ± 0.02	0.82 ± 0.03*
*Night period*		
VO_2_ (mL/min)	8.31 ± 0.39	7.36 ± 0.26 **
VCO_2_ (mL/min)	7.12 ± 0.18	53.15 ± 1.34 **
EE (kcal/day)	59.49 ± 1.39	6.45 ± 0.37 *
RQ	0.86 ± 0.01	0.81 ± 0.02 *

Mean values ± SEM. *VO*_2_ O_2_ consumption, *VCO*_2_ CO_2_ production, *EE* energy expenditure, *RQ* respiratory quotient; * Significantly different (*p* < 0.01) from the control group by Student’s *t*-test. ** Significantly different (*p* < 0.001) from the control group by Student’s *t*-test.

**Table 6:** Plasma concentration of hormones affecting the basal metabolic rate.

	Normal-Fe Control Group (*n* = 20)	Low-Fe Anaemic Group (*n* = 20)
TSH (pg/mL)	29.43± 3.96	51.32 ± 5.21 **
T_3_ (pg/mL)	15,269.21 ± 432.21	10,123.29 ± 348.17 **
T_4_ (pg/mL)	1422.45 ± 98.77	1098.32 ± 95.37 **
Ghrelin (pg/mL)	36.42 ± 1.05	14.45 ± 0.81 **
GIP (pg/mL)	26.98 ± 1.24	33.28 ± 1.65 *
Glucagon (pg/mL)	24.23 ± 1.13	30.18 ± 1.24 *
Insulin (pg/mL)	726.43 ± 32.18	862.22 ± 29.98 *
Corticosterone (ng/mL)	177.65 ± 27.45	341.65 ± 41.24 **
ACTH (pg/mL)	1252.11± 109.23	861.32± 87.54 **

* Significantly different (*p* < 0.01) from the control group by Student’s *t*-test. ** Significantly different (*p* < 0.001) from the control group by Student’s *t*-test. TSH Thyroid-stimulant hormone, GIP Gastric inhibitory polypeptide, ACTH Adrenocorticotropic hormone.
